# The Relationship Between Cytokeratins 7 and 20 Expression, and Prognostic Factors in Colon Adenocarcinoma: An Immunohistochemical Study

**Published:** 2017-04-01

**Authors:** Mohammad Hossein Gheini, Noushin Jalayer Naderi

**Affiliations:** 1 *Dept. of Pathology, Faculty of Medicine, Shahed University, Tehran, Iran*; 2 *Dept. of Oral and Maxillofacial Pathology, Faculty of Dentistry, Shahed University, Tehran, Iran*

**Keywords:** Cytokeratin 7, Cytokeratin 20, Colon Adenocarcinoma, Prognosis

## Abstract

**Background & Objective:**

The role of synchronized expression pattern of cytokeratin (CK) 7 and CK20 in the prognosis of colon adenocarcinoma is unclear. The current study aimed at determining the relationship between the expression of cytokeratins 7 and 20 and prognostic factors in colon adenocarcinoma.

**Methods:**

In the current cross sectional Study, 52 archival samples of colon adenocarcinoma with different histopathologic differentiation were examined immunohistochemically to analyze the expression of Ck7 and Ck20. The relationship between cytokeratin expression and prognostic factors, such as histopathologic differentiation, lymph node involvement, and depth of invasion, were assessed.

**Results:**

CK7-/CK20+ was the most prevalent pattern in the current study. The difference among histopathologic grade, lymph node involvement, and depth of invasion in different CK7/CK20 expression patterns was insignificant (P=0.26, P=0.46, and P=0.22, respectively).

**Conclusion:**

No relationship was observed between CK7/CK20 expression and prognostic factors in colon adenocarcinoma, in the current study.

## Introduction

Colorectal carcinoma is the most common malignancy of gastrointestinal tract. It is the second cause of death in females and third in males. The patients who survive colorectal carcinoma are prone to metachronous diseases and recurrence (1).

The clinicopathological staging is not enough to determine the outcome of malignancy. Prognosis is the most important factor in patients` care and survival in colorectal carcinoma (2).

 To determine the prognosis, it is necessary to have a reliable tool. Immunohistochemical analysis is the most common and viable employed application. Cytokeratins (CKs) are intermediate filaments found in the cytoskeletal component of epithelial cells. Different profiles of CKs are expressed in malignant tissues with epithelial origin. CKs consist of 20 subtypes. The expression of CKs mainly depends on epithelial cell type and differentiation (3).

The expression of CK7 and CK20 are shown in colorectal carcinoma. The main pattern of CK expression in colorectal carcinoma is CK7–/CK20+ (4-5).

CK7 is the intermediate filament that consists of the gland and transitional epithelium. This type of CK is not found in squamous epithelium (6).

CK20 is a particular type of CKs observed on gastric and colon adenocarcinoma. For the first time, CK20 was diagnosed on gastric and intestinal epithelium (7).

CK7 and CK20 are the most useful cytokeratins to diagnose and differentiate carcinomas. The co-expression of CK7 and CK20 is examined in different carcinomas (8-10).

Recently, researches have focused on detecting the origin of metastatic carcinomas, using the CK7 and CK20 (11-13).

In spite of such efforts, the role of synchronized expression pattern of CK7 and CK20 in relation to prognosis is not clear. 

The current study aimed at determining the relationship between expression of Ck7 and Ck20, and prognostic factors in colonadenocarcinoma.

## Material and Methods

The current cross sectional study employed the descriptive-analytical method. Tissue sampling was based on archive. The pathologic records of colon adenocarcinoma were retrieved from the archive of pathology department, Shahid Mostafa Khomini Hospital, Tehran, Iran, from 2008 to 2014. The hematoxylin-eosinstained slides were reviewed. The samples with sufficient tissue material and complete medical record information were selected. Adequate tumoral mass, absence of necrosis/hemorrhage, presence of lymph node pathologic slides, and complete medical records were the inclusion criteria. Based on the inclusion criteria, 52 formalin-fixed, paraffin-embedded samples were selected. Demographic and clinical information including age, gender, depth of tumor, number of involved lymph nodes, and histopathologic grade were registered. Samples were classified in 4 groups: Cytokeratin7+/cytokeratin20+ (CK7+/20+), cytokeratin7+/cytokeratin20- (CK7+/20-), cytokeratin7-/cytokeratin20+(CK7-/20+), and cytokeratin7-/cytokeratin20- (CK7-/20-).

**Immunohistochemical Analysis**

The 4-μm paraffinized sections were soaked in water-alcohol solution for 5 minutes. Slides were placed in microwave oven for 30 minutes at 60°C. Deparaffinization was completed by soaking the slides in xylene and, then, alcohol (from 100% to 75% concentration) for 5 to 10 minutes. Sections were rinsed with 10% phosphate-buffered saline (PBS) followed by H_2_O_2_/methanol (1:9) and 10% PBS for 10 minutes. Then, the slides were heated in microwave oven for 10 minutes in ethylenediaminetetraacetic acid (EDTA). The samples were left to reach the room temperature; then, were rinsed with PBS. Sections were incubated with 1 μg/mL diluted primary anti-mouse monoclonal antibody for 1 hour at room temperature (Novocastra Ltd., England) and, then, were reincubated with biotinylated antibody for 30 minutes and soaked in 10%PBS for 10 minutes. Sections were incubated with conjugated enzyme for 30 minutes and developed in 3, 3`-diaminobenzidinehydrochloride (DAB).

Haematoxylin stain was used to develop the ground contrast. Slides were passed in distilled water-alcohol from 75% to 100% for 3 to 5 minutes and before mounting were immersed in xylene for 5 minutes. Between incubations, all samples were rinsed with PBS. The small intestine and cerebellum were used as positive and negative controls, respectively. 

The expression of CK7 and CK20 were examined by light microscopy (Olympus CH-2, Japan) at ×400 magnification. The scoring was blind and performed by an experienced general pathologist. The cytokeratin expression was obtained by the percent score of total count of malignant cells/total count of stained cells in 5 microscopic fields (14).

 For statistical analysis, the ANOVA, Mann-Whitney, and Duncan tests were employed using SPSS 19 software. P <0.05 was considered as statistical significance level.

## Results

There were 29 (55.76%) males and 23 (44.24%) females in the current study samples. The mean age was 61 years. The epithelial cells with brown-stained cytoplasm were considered positive for CK 7 and CK 20 expression.

The total number of 47 (90.38%) cases was CK20+, followed by 41(78.85%) CK 7-, 11(21.15%) CK 7+, and 5 (9.62%) CK 20-.

The total number of 39 (75%) cases was CK7-/CK 20+, followed by 8(15.39%) CK 7+/CK20+, 3 (5.77%) CK 7+/CK20-, and 2 (3.84%) CK7-/CK20-.

**Table 1:** CK7/CK20 Expression and Clinicopathological Findings in Colon Adenocarcinoma

CK Expression	CK20+, n (%) 47(90.38)	CK20-, n (%) 5(9.62)	CK7+, n (%) 11(21.15)	CK7-, n (%) 41(78.85)
Low grade	32(68.08)	2(40)	4(36.36)	30(73.17)
High grade	15(31.92)	3(60)	7(63.64)	11(26.83)
Lymph Nodes+	24(51.06)	33(60)	8(72.73)	19(46.34)
Lymph Nodes-	23(48.94)	2(40)	3(27.27)	22(53.66)
Full involvement	36(76.60)	4(80)	7(63.64)	33(80.49)
Partial involvement	11(23.40)	1(20)	4(36.36)	8(19.51)

**Table 2:** CK7/CK20 Expression Patterns and Clinicopathological Findings in Colon Adenocarcinoma

CK Pattern	CK7-/CK20+, n (%) 39(75)	CK7+/CK20+, n (%) 8(15.39)	CK7-/CK20-, n (%) 2(3.84)	CK7+/CK20-, n (%) 3 (5.77)	Total, n (%) 52 (100)
Low grade	29 (55.79)	3 (5.77)	1 (1.92)	1(1.92)	34 (65.38)
High grade	10(19.23)	5(9.62)	1(1.92)	2(3.85)	18 (34.62)
Lymph Nodes+	18(34.62)	6(11.54)	1(1.92)	2(3.85)	27(51.93)
Lymph Nodes-	21(40.38)	2(3.85)	1 (1.92)	1(1.92)	25(48.07)
Full involvement	31(59.61)	5(9.62)	2(3.85)	2(3.85)	40(76.93)
Partial involvement	8(15.39)	3(5.77)	0	1(1.92)	12(23.07)

CK, cytokeratin

The difference among histopathologic grade, lymph node involvement, and depth of invasion in different CK20/CK7 expression patterns was insignificant (P=0.26, P=0.46, and P=0.22, respectively).

**Fig 1:**
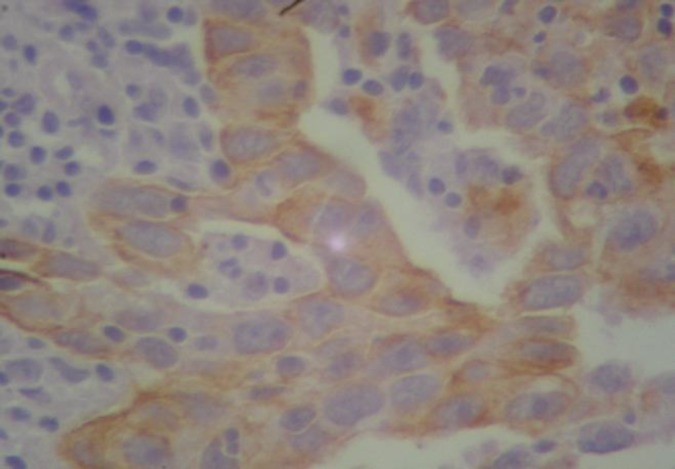
Immunohistochemical staining of cytokeratin 7 expression in colon adenocarcinoma. The strong immunoreactivity in the cytoplasm of colon adenocarcinoma cells (x400)

## Discussion

The results of the current study showed no relationship between CK 7 and CK 20 expression patterns and prognostic factors in colon adenocarcinoma.

**Fig 2:**
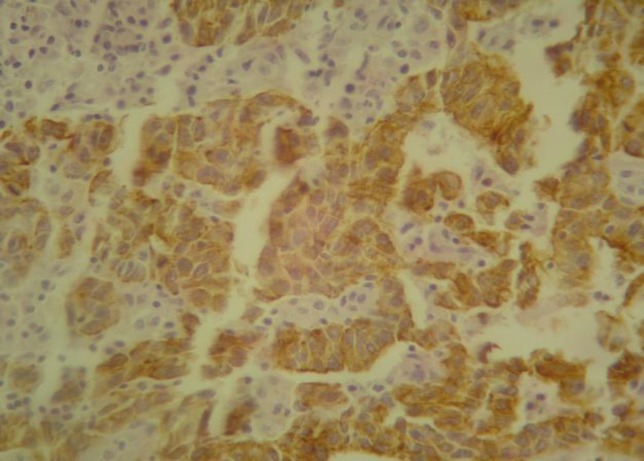
Immunohistochemical staining of cytokeratin 20 in colon adenocarcinoma. The colon adenocarcinoma cells showed strong positive CK20 immunostaining (x400)

The different expression patterns of CK 7 and CK 20 in tumors with epithelial origin is a useful tool in differential diagnosis of carcinomas (9, 11-17).

Most efforts concentrate on CK7 and CK 20 expression in different metastatic carcinomas to detect the site of their origin. However, the existing data regarding the correlation of the CK expression pattern with clinicopathological parameters are rare.

Based on reports, the most prevalent pattern of CK 7 and CK 20 expression in colon adenocarcinomas was CK 7-/CK20+ (5-8-18).

It was consistent with the present study results, which showed that CK7-/CK20+ pattern was more common. However, the CK7-/CK20+ expression pattern was not shown in all colorectal carcinomas.

The current study examined the relationship between the expression of CK7/CK20 patterns, and histopathologic grade, lymph node involvement and depth of invasion in colon adenocarcinoma. The different CK 7 and CK 20 expression patterns were not related to clinicopathological features in colon adenocarcinomas. The current study finding was inconsistent with some previous reports.

Bayrak et al., reported that the CK7 and CK20 expression patterns depend on histological grade, location of the tumor, and lymph node metastasis. They showed that CK7 expression was more prevalent in tumors with lymph node metastasis. On the other hand, CK20+ was more common in low grade proximal colon carcinomas (88.9%) (18).

Park et al., in a retrospective study showed that all of the 225 cases of metastatic colorectal carcinoma to the ovary were CK7-/CK20+ and only one case was CK7-/CK20-; 75% of low-grade and 52% of high-grade carcinomas were CK7-/CK20+ (5).

In the present study, the difference among histopathologic grade, lymph node involvement, and depth of invasion in different CK 7 and CK 20 expression patterns was insignificant. Some existing disagreements between the current results and those of the previous studies may be due to different sampling. In the present study, all samples were collected from patients with colon adenocarcinoma. However, in prior reports patients with colorectal carcinomas were included for examination. It was shown that the CK7 and CK20 expression patterns were different according to the location of the tumor in the colorectal region (18). 

Based on the results of the study, the expression pattern of CK7/CK20 was a useful tool for the differential diagnosis of colon adenocarcinoma. However, it seems that different expression patterns of CK7/CK20is not valuable to determine the prognosis in colon adenocarcinoma. More studies considering genetic differences and different degrees of differentiation are necessary. No previous reports indicated the relationship between CK7 and CK20 expression patterns and clinicopathological parameters in colon adenocarcinoma. Due to this pitfall, further studies should be conducted.

## Conclusion

There was no relationship between the patterns of CK7/CK20 expression and prognostic factors in colon adenocarcinoma.
